# Activation of *Six1* Expression in Vertebrate Sensory Neurons

**DOI:** 10.1371/journal.pone.0136666

**Published:** 2015-08-27

**Authors:** Shigeru Sato, Hiroshi Yajima, Yasuhide Furuta, Keiko Ikeda, Kiyoshi Kawakami

**Affiliations:** 1 Division of Biology, Center for Molecular Medicine, Jichi Medical University, Shimotsuke, Tochigi, Japan; 2 Animal Resource Development Unit and Genetic Engineering Team, Division of Bio-function Dynamics Imaging, RIKEN Center for Life Science Technologies (CLST), Kobe, Hyogo, Japan; 3 Division of Biology, Hyogo College of Medicine, Nishinomiya, Hyogo, Japan; Universitat Pompeu Fabra, SPAIN

## Abstract

SIX1 homeodomain protein is one of the essential key regulators of sensory organ development. *Six1*-deficient mice lack the olfactory epithelium, vomeronasal organs, cochlea, vestibule and vestibuloacoustic ganglion, and also show poor neural differentiation in the distal part of the cranial ganglia. Simultaneous loss of both *Six1* and *Six4* leads to additional abnormalities such as small trigeminal ganglion and abnormal dorsal root ganglia (DRG). The aim of this study was to understand the molecular mechanism that controls *Six1* expression in sensory organs, particularly in the trigeminal ganglion and DRG. To this end, we focused on the sensory ganglia-specific *Six1* enhancer (Six1-8) conserved between chick and mouse. *In vivo* reporter assays using both animals identified an important core region comprising binding consensus sequences for several transcription factors including nuclear hormone receptors, TCF/LEF, SMAD, POU homeodomain and basic-helix-loop-helix proteins. The results provided information on upstream factors and signals potentially relevant to *Six1* regulation in sensory neurons. We also report the establishment of a new transgenic mouse line (mSix1-8-NLSCre) that expresses Cre recombinase under the control of mouse Six1-8. Cre-mediated recombination was detected specifically in ISL1/2-positive sensory neurons of *Six1*-positive cranial sensory ganglia and DRG. The unique features of the mSix1-8-NLSCre line are the absence of Cre-mediated recombination in SOX10-positive glial cells and central nervous system and ability to induce recombination in a subset of neurons derived from the olfactory placode/epithelium. This mouse model can be potentially used to advance research on sensory development.

## Introduction

*Sine oculis-related homeobox 1* (*Six1*) is a member of the *Six* homeobox gene family [1, 2]. In humans, mutations of *SIX1* cause branchio-oto-renal (BOR) syndrome, an autosomal dominant disorder characterized by hearing loss, branchial arch defects and various kidney abnormalities [3, 4]. Mice deficient in *Six1* show severe defects in diverse cranial sensory organs and neurons. They lack sensory organs/neurons derived from the olfactory placode (olfactory epithelium, vomeronasal organ and gonadotropin-releasing hormone neurons) and the otic placode (cochlea, vestibule and vestibuloacoustic ganglion), and show reduced neural differentiation in the distal portion of the epibranchial ganglia derived from the epibranchial placodes [5–10]. The simultaneous loss of both *Six1* and the adjacent *Six4* aggravates these defects, and leads to additional defects such as minute trigeminal ganglion and abnormal dorsal root ganglia (DRG) characterized by the presence of immature neurons and fusion between adjacent ganglia at the lumber region [9, 11, 12]. A study using *Xenopus* also revealed that SIX1 is required for both the regulation of neuronal progenitor proliferation and subsequent neuronal differentiation [13]. Neurogenesis in the mouse olfactory epithelium is also dependent on *Six1* [6, 9, 10]. Thus, SIX1 is assumed to be one of the essential key regulators of the development of sensory organs and neurons.

In the ectoderm of tetrapods, *Six1* is initially expressed in a precursor domain common for all cranial placodes, termed the preplacodal region (PPR). Furthermore, the gene is expressed in all neurogenic placodes [olfactory, trigeminal, otic, epibranchial and lateral line (in *Xenopus* and zebrafish) placodes] and the adenohypophyseal placode [14–19], and is persistently noted in the cranial sensory organs/ganglia and the Rathke's pouch/adenohypophysis. In the trunk, the gene is expressed in the DRG [11, 17, 20, 21], specifically in the sensory neurons [11]. In the chick and mouse, the expression of *Six1* during embryogenesis is controlled by at least eight conserved enhancers [11, 17, 22, 23]. The molecular mechanisms involved in the regulation of *Six1* were previously discussed after analyzing Six1-14, the sole PPR enhancer, and Six1-21, one of the four placode enhancers [17, 23, 24]. However, *Six1* regulation in sensory ganglia, such as the trigeminal placode/ganglion and the DRG, is yet to be analyzed. Also, apart from the information on enhancers and target genes of *Brn3a* and *Isl1* [25–28], our knowledge concerning specific enhancers and regulatory hierarchy among transcription factors relevant to the early development of the two sensory ganglia is limited. *Six1* expression in the trigeminal placode/ganglion is controlled by Six1-8, a major conserved enhancer, and Six1-9, a weak amniote-specific enhancer [17]. Six1-8 also activates transcription in the otic placode/pit/vesicle, epibranchial placodes/ganglia and DRG [17]. In the trunk region of *Xenopus* larva, Six1-8 activates transcription in a transient population of intramedullary mechanosensory neurons termed the Rohon-Beard (RB) cells, in addition to the DRG [11, 29].

The aim of the present study was to provide a better understanding of the molecular mechanism that controls *Six1* expression in the sensory organs particularly the trigeminal ganglion and the DRG. Functional analysis of Six1-8 identified an important core region that contains the binding consensus sequences for various upstream factors and signals. Based on the findings, we report the establishment of a new transgenic mouse line that expresses Cre recombinase in sensory neurons in the cranial sensory ganglia and the DRG under the control of Six1-8.

## Materials and Methods

### Animals

Fertilized eggs of chicken were purchased from Shiroyama Poultry Farm (Kanagawa, Japan), and incubated at 38°C in a humidified rocking incubator. The developmental stage of chick embryos was determined by the Hamburger and Hamilton (HH) stages [30]. Mice were housed in an environmentally-controlled room at Jichi Medical University and the RIKEN CDB under the guidelines for animal experiments as described previously [17, 23]. All animal experiments were carried out in a humane manner after receiving ethical approval of the Institutional Animal Experiment Committee of the Jichi Medical University, and in accordance with the Institutional Regulation for Animal Experiment and Fundamental Guideline for Proper Conduct of Animal Experiment and Related Activities in Academic Research Institutions under the jurisdiction of the MEXT of Japan.

### Genomic sequence analysis

The genomic sequences containing *Six1* were downloaded from Ensembl [31]: Mouse (GRCm38.p3, INSDC Assembly, Jan 2012), Human (GRCh38, INSDC Assembly Dec 2013), Chicken (Galgal4, INSDC Assembly, Nov 2011), *Xenopus* (JGI 4.2, INSDC Assembly, Nov 2009), coelacanth (LatCha1, INSDC Assembly, Sep 2011), medaka (HdrR, Oct 2005) and spotted gar (LepOcu1, INSDC Assembly, Dec 2011). Global pairwise alignment was carried out using shuffle-LAGAN [32], and the results were visualized using the VISTA Browser [33]. Conserved transcription factor binding consensus sequences were identified using rVISTA [34] and visual inspection of the aligned sequences.

**Fig 1:**
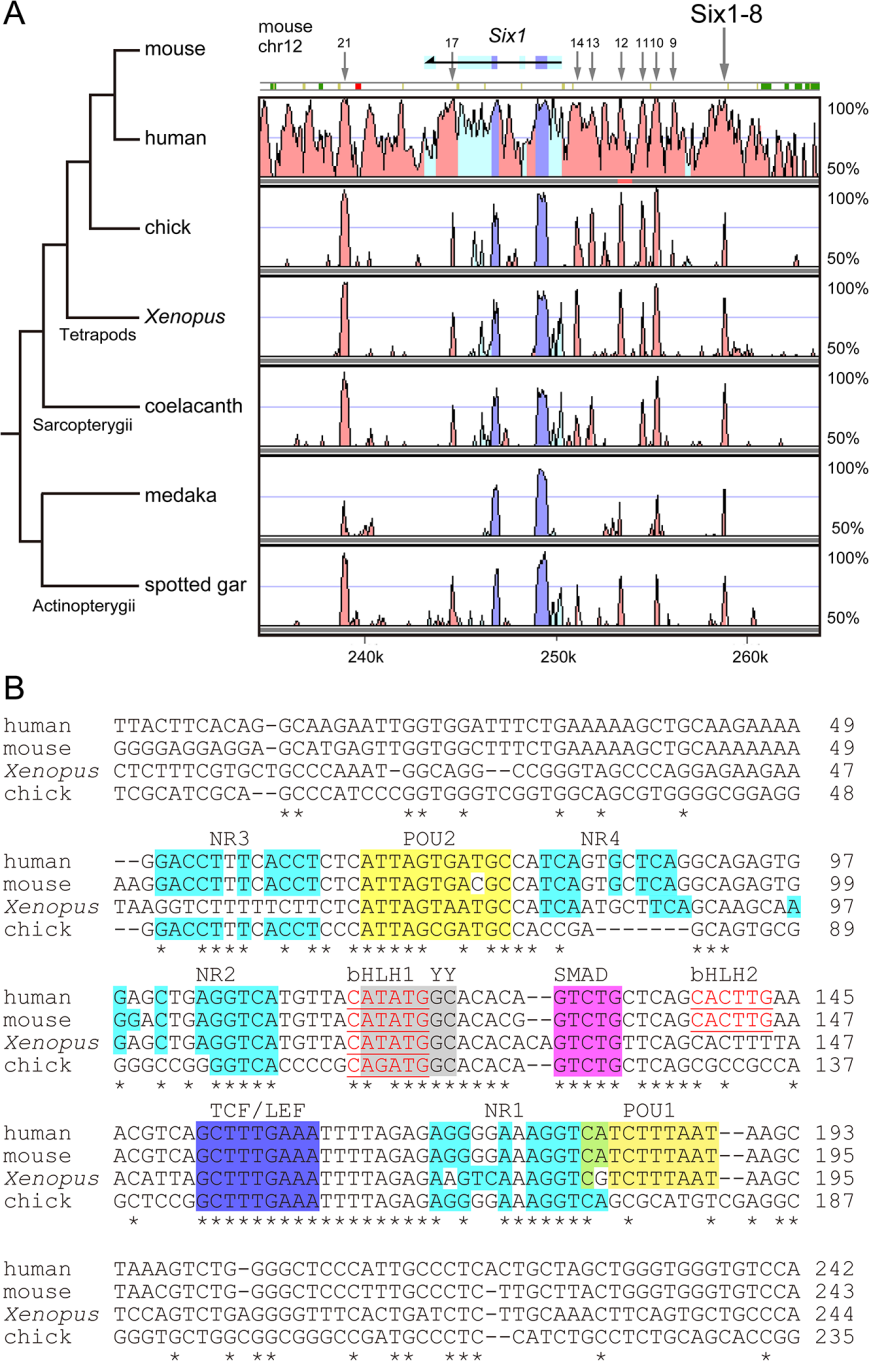
Sequence characteristics of Six1-8 enhancer. (A) The VISTA plot of the 29-kb interval containing mouse *Six1*. The plot shows conserved sequences between mouse and human, chicken, *Xenopus*, coelacanth, medaka and spotted gar. Horizontal axis: mouse sequence, vertical axis: percentage identity in a 50-bp window. The conserved regions with >60% identity over 50 bp are highlighted under the curve. *Pink*: conserved non-coding sequence (CNS), *blue*: conserved exon, *cyan*: untranslated region. Six1-8 (indicated by large arrow) located 9-kb upstream of *Six1* exon1 is conserved in all seven species. The positions of eight other conserved *Six1* enhancers (Six1-9, 10, 11, 12, 13, 14, 17 and 21) are also indicated by small arrows. The phylogenetic relationship of the seven species is shown on the left. Numbers at the bottom of the plot indicate positions relative to the analyzed 500-kb mouse genome fragment (chromosome:GRCm38:12:72796952:73296951:1). (B) Alignment of the core conserved regions of Six1-8 from human, mouse, *Xenopus* and chick. Binding consensus sequences for nuclear receptors (NR1-NR3, an additional site similar to NR is indicated as NR4, cyan shading), BRN2/3 (POU1 and POU2, yellow shading), YY1 (YY, gray shading), SMAD proteins (magenta shading), TCF/LEF (dark blue shading), and bHLH protein-binding E-boxes (bHLH1 and bHLH2, underlined red letters). (*) identical bases, (-) gaps introduced to maximize similarity. Guanine (G) at the position [chromosome:GRCm38:12:73055589 on mouse genome] and corresponding bases of other species are numbered one (+1). The orientation of the aligned sequences is opposite to *Six1* transcription (moue *Six1*: chromosome:GRCm38:12:73041827:73047179:-1).

In previous studies, we identified eight *Six1* enhancers (Six1-8, 9, 10, 11, 12, 14, 17 and 21) among 16 evolutionarily conserved sequences, using *in vivo* enhancer analysis in chick and mouse [17, 23]. During the execution of the study, the conserved 572-bp sequence (Six1-13) of mouse origin was identified as a cardiac-specific enhancer by another group [22] and was thus included in Fig 1 as Six1-13.

### Construction of reporter plasmid

ptkmRFP1 mSix1-8wt was constructed by transferring a 538-bp wild-type mouse Six1-8 fragment (mSix1-8) from ptkEGFP-mSix1-8wt [17] to ptkmRFP1 [35, 36]. Mutated ptkEGFP-mSix1-8 reporters were constructed using oligonucleotide primes designed based on earlier studies [37–46] (listed in Table 1) by PCR as described previously [17]. The ASSinsBBins-mSix1-8-tkintronLacZpA used for mouse transgenesis was reported previously (tkintron: the thymidine kinase gene promoter of HSV and the downstream chimeric intron, pA: SV40 polyA region [17]). The HS4 insulator (ins) from the chicken ß-globin locus [47] is essential to obtain reproducible results. Both ptkEGFP-mSix1-8NR1-4m and ptkEGFP-mSix1-8TCF/LEFm2 were used to prepare mutated LacZ reporter plasmids for mouse transgenesis.

**Table 1:** PCR primers used to introduce mutations in mSix1-8.

Primer name	Sequence	Introduced mutation	References
8-COUP1m	TTTAGAGAGGcaAAAaacCATCTTTAATAAG	NR1m, NR1-4m	[37–39]
8-COUP2m	CAGAGTGGGcaaGAaacCATGTTACATAT	NR2m, NR1-4m	[37–39]
8-COUP-3m	TTAGTGACGCCAcaAGTaacCAGGCAGAGTGGGA	NR4m, NR1-4m	[37–39]
8-COUP4m2	GCAAAAAAAAAGGgttTTgttCCTCTCATTAGTGAC	NR3m, NR1-4m	[37–39]
8-LEF1m2	CTTGAAACGTCAaaTTTcAAATTTTAGAGAGGGG	TCF/LEFm2	[40]
Six1-8-Smad-1	ATATGGCACACGGTtTGCTCAGCACTTGA	SMADm	[41, 42]
Six1-8-Smad-2	TCAAGTGCTGAGCAaACCGTGTGCCATAT	SMADm	[41, 42]
Six1-8-Ebox-m3-1	TGAGGTCATGTTAaAaATGGCACACGGTC	bHLH1m, bHLH12m	[43]
Six1-8-Ebox-m3-2	GACCGTGTGCCATtTtTAACATGACCTCA	bHLH1m, bHLH12m	[43]
8-Ebox2-m1	GTCTGCTCAGCggccGAAACGTCAGCTTT	bHLH2m, bHLH12m	[43]
8-POU-1m	GGAAAGGTCATCTTTgcTAAGCTAACGTCTGGG	POU1m, POU12m	[44]
8-POU-2m (8-1-POU-m1)	CCTTTCACCTCTCAgcAGTGACGCCATCAGT	POU2m, POU12m	[44]
Six1-8-YY1-3	CATGTTACATATGaaAaACGGTCTGCTCAG	YYm	[45, 46]
Six1-8-YY1-4	CTGAGCAGACCGTtTttCATATGTAACATG	YYm	[45, 46]
pGL3	CTAGCAAAATAGGCTGTCCC	universal primers used in LA PCR mutagenesis system	[17, 23]
ptkEGFP-RP	GGACACCGCCAGCAAACG	universal primers used in LA PCR mutagenesis system	[17, 23]
ptkEGFP-mutBX	TTAGATCGCAGATCaCGAGCC	universal primers used in LA PCR mutagenesis system	[17, 23]

Positions corresponding to the predicted transcription factor binding sites are underlined. Mutated nucleotides are in lower case.

In order to detect even a weak enhancer activity in electroporated chick embryos, stable integration of the reporter construct into the chromosome should be advantageous [48]. Therefore, we constructed pT2A-BB-mSix1-8-EGFP containing the medaka Tol2 element from pT2AL200R150G [49]. First, an annealed XBB oligonucleotides (XBB-top: 5'-tcgagCGTACGTCGACa, XBB-bottom: 5'-gatctGTCGACGTACGc, partial-*Xho*I and partial-*Bgl*II sites at the 5' and 3' ends are in lowercase, *Bsa*WI site are underlined) were inserted into the *Xho*I-*Bgl*II sites of pT2AL200R150G, replacing its EGFP expression unit. Then, the resultant pT2A-BB was cut with *Bsa*WI and *Bgl*II, and ligated with the mSix1-8-tkintron-EGFP cassette (as a *Bsa*WI-*Bgl*II compatible *Acc*65I-*Bam*HI fragment) from ptkEGFP-mSix1-8wt.

### Reporter assay in chick embryos

Chick embryo electroporation was carried out as described previously [17]. HH4-5 embryos were used to introduce DNA into the entire epiblast and assess enhancer activity in the cranial region after culture on albumen-agar plate. HH14 embryos were used to introduce DNA into the neural tube (left side) in ovo and examine enhancer activity in the DRG. CUY21EDIT electroporator (BEX Co., Japan) was used with a pair of 2 x 2 mm square platinum plates (HH4-5 electroporation: 5 v/4 mm, 50 ms pulse, 50 ms interval, 5 pulses) and a pair of platinum electrodes with 0.5 mm diameter [HH14 electroporation: 100 v/3 mm, 0.1 ms pulse (pre-pulse), followed by 0.1 ms interval, and 15 v/3 mm, 50 ms pulse, 50 ms interval, 5 pulses]. The DNA solution used for mSix1-8 mutation analysis contained 1.78 mg/ml of ptkmRFP1-mSix1-8wt control plasmid and 2.22 mg/ml of ptkmEGFP-mSix1-8 mutated reporters and 0.02% Fast Green in 1x TE [10 mM Tris-HCl (pH 7.5), 1 mM EDTA]. In Tol2 experiment, the DNA solution containing 2.0 mg/ml of pT2A-BB-mSix1-8wt, 0.8 mg/ml pCS-TP [50] and 0.02% Fast Green in 1x TE (pH 7.5) was introduced into the entire epiblast at HH4-5. The embryos were examined at 48 hours post-electroporation (h.p.e.) using a stereomicroscope (M205A, Leica Microsystems, Wetzlar, Germany).

For quantification, the EGFP and mRFP1 images of each embryo were acquired using the same exposure time that does not saturate pixels in both channels (other conditions such as detector gains were kept constant). To assess the effects of various mutations on mSix1-8 enhancer activity in the DRG, the relative fluorescence intensity was determined by measuring five ganglia on the left side (in whole mount dorsal images). To assess the effects in the cranial ganglia, the relative fluorescence intensity of trigeminal ganglia on both sides (in whole mount lateral images, excluding axons extend from the ganglion) was measured. The mean EGFP level normalized to the mRFP1 level (after subtracting background) was computed for each embryo, and more than five embryos were measured for each mutation. In the figures, all values are expressed as mean ± standard deviation. Differences from the wild-type reporter were evaluated with Student’s t-test. A probability of less than 5% was considered statistically significant.

### Construction of a transgene for Cre recombinase expression

mSix1-8 was used to construct a transgene that drives the expression of Cre recombinase of P1 bacteriophage in the SIX1-positive sensory neurons/ganglia. The coding sequence of the Cre recombinase gene was amplified from the plasmid pIGCN21 [51] with a pair of primers, Cre-NLS-FP (5'-agct **c** **cc aag aag aag agg aag gtg** TCC AAT TTA CTG ACC GTA C, partial *Sac*I site at the 5' end is underlined, Cre coding sequence is in uppercase) and Cre-XbaI-RP (5'-tatatatctaga CTA ATC GCC ATC TTC CAG CAG G, *Xba*I site is underlined, Cre coding sequence is in uppercase), which are designed to add the nuclear localization signal (NLS) of SV40 large T antigen (PKKKRKV, corresponding to bold lowercase sequence of Cre-FP) at the N-terminus. The NLSCre PCR fragment was phosphorylated, blunted, cut with *Xba*I and ligated into the *Sac*I(blunted)-*Xba*I sites of the ptkEGFP (ptkEGFP-partial-NLSCre). Then, the mSix1-8-tkintron cassette from ptkmRFP1-mSix1-8wt [as a *Kpn*I-*Nco*I (blunted) fragment] was inserted into the *Kpn*I-*Sac*I (blunted) sites of ptkEGFP-partial-NLSCre. The resultant plasmid was cut with *Sal*I, and a *Sal*I-fragment containing mSix1-8-tkintron-NLSCre linked to the downstream SV40 polyA region was ligated into the *Bsm*BI site (compatible with *Sal*I ends) of the ASSinsBBins vector [17] to add HS4 insulators to both ends.

### DNA sequencing and purification

The DNA sequences of all plasmids were verified by chain termination sequencing and the plasmids were purified using the QIAfilter Plasmid Midi Kit (for chick electroporation, Qiagen, Hilden, Germany) or EndoFree Plasmid Maxi Kit (for mouse transgenesis, Qiagen). All transgenes for mouse microinjection were excised from ASSinsBBins vectors as *Sal*I- or *Not*I-fragments (5.7 kb fragment, for transient transgenesis) or a *Kpn*I(5')-*Sac*II(3') fragment (3.7 kb fragment, for mSix1-8-NLSCre line), run on an agarose gel and purified using QIAEX II gel extraction kit (Qiagen). Table 2 lists all plasmids used in this study.

**Table 2:** Plasmids used in this study.

Plasmids	Reference
ptkEGFP constructs	
mSix1-8wt	[17]
mSix1-8NR1m	
mSix1-8NR2m	
mSix1-8NR1-4m	
mSix1-8TCF/LEFm2	
mSix1-8SMADm	
mSix1-8bHLH12m	
mSix1-8POU12m	
mSix1-8YYm	
ptkmRFP1 construct	
mSix1-8wt	
ASSinsBBins constructs	
mSix1-8-tkintronLacZpA	[17]
mSix1-8NR1-4m-tkintronLacZpA	
mSix1-8TCF/LEFm2-tkintronLacZpA	
mSix1-8-tkintron-NLSCre	
pT2ABB construct	
mSix1-8-tkintron-EGFP	
pCS-TP	[50]

### Transient transgenesis of mouse embryos

Transient transgenesis of mouse embryos was carried out using fertilized eggs of the CD-1 (ICR) strain, as described previously [17, 23]. Transgenic embryos were dissected at E10.5 and assessed for ß-Galactosidase (ß-Gal) activity, and the yolk sacs were processed for genotyping using Direct PCR Lysis Reagent (Viagen Biotech, Los Angeles, CA) [17, 23]. The yolk sac DNAs were subjected to PCR with a pair of primers, mSix1-8-3 (5’-GCAGATGAAAGGCAGGGCTACT) and ptkEGFP-RP (5'-GGACACCGCCAGCAAACG), to identify embryos carrying LacZ reporter transgenes.

### Production and genotyping of transgenic mouse line

Transgenic mice that harbor mSix1-8-tkintron-NLSCre-ins transgene were produced by a standard protocol [52] using fertilized eggs of the BDF1 strain. Seven potential founder mice were identified from 44 live births by PCR analysis of tail-tip DNA with a pair of primers specific to Cre, Cre-PCR1 (5'-TGCCAGGATCAGGGTTAAAGAT) and Cre-PCR2 (5'-AGCTTGCATGATCTCCGGTATT), which produces a 409-bp product. Also, integration of the transgene was confirmed by Southern blotting/hybridization using the 1,577-bp *Bgl*II-*Not*I fragment that covers the tkintron-Cre region as a probe. Then, the seven founder mice (lines 22L, 30L, 31L, 32L, 33L, 7l and 35l) were crossed with C57BL/6J mice, and their offsprings (F1 and F2 generations) were used to detect Cre-mediated recombination. Each transgenic male offspring was crossed with R26R-LacZ females [53], embryos were collected at stages E9.0–12.5 and stained for ß-Gal activity, as described previously [17, 23]. mSix1-8-NLSCre/R26R-LacZ double transgenic embryos were identified by PCR using two primer pairs, Cre-PCR1/2 and 8114-lacZ-3 (5'-ACTATCCCGACCGCCTTACT)/8114-lacZ-4 (5'-TAGCGGCTGATGTTGAACTG). The 35l line showed sensory ganglia-specific recombination, and the line has been maintained as heterozygous by backcrossing onto C57BL/6J for 17 generation (currently, N17). Cre-PCR1/2 primers were used for routine tail-tip genotyping. The 35l line (mSix1-8-NLSCre, Accession No. CDB0507T: http://www.clst.riken.jp/arg/TG%20mutant%20mice%20list.html) will be deposited to the RIKEN BioResource Center (BRC).

On the C57BL/6J background, some transgenic embryos showed ubiquitous Cre activity superimposed on the aforementioned specific pattern even at N16 generation. This ectopic Cre activity usually appear at similar stage as the specific one, and the level of ectopic activity varied among embryos. Based on examples of several transgenic lines where Cre excision varied from tissue specific to ubiquitous within a single litter, such inconsistent Cre activity was considered to represent an intrinsic property of the gene promoter/enhancer driving Cre [54, 55]. It is possible that one of the characteristics of isolated mSix1-8 is a sporadic burst of nearly ubiquitous expression at around the onset of activation specific to sensory ganglia.

### Histochemistry and immunofluorescence

To detect ß-Gal, mouse embryos at E9.0–12.5 were fixed with 4% paraformaldehyde [in phosphate buffered saline (PBS)] for 15 min at room temperature and processed for X-Gal staining, as described previously [17, 23]. After staining, the embryos were washed with PBS, refixed with 4% paraformaldehyde for 75 min. Other chick and mouse embryos were fixed with 4% paraformaldehyde for 120 min at room temperature. For histological analysis, embryos were washed with PBS, immersed in 30% sucrose/PBS, embedded in optimal cutting temperature (OCT) compound (Sakura Finetek), then frozen on dry ice, and cut into 14- or 16-μm thick sections. The images of ß-Gal staining were obtained with a stereomicroscope (SZX16, Olympus, or M205A, Leica Microsystems) and a standard microscope (BX51, Olympus, or DM5000B, Leica Microsystems).

Immunofluorescence was carried out as described previously [6, 11, 17, 56]. The following primary antibodies were used: rabbit anti-ß-Gal (dilution, 1:5000, Covance, Berkeley, CA), mouse anti-ISL1/2 (dilution, 1:150, mixture of hybridoma supernatants, 39.4D5 and 40.2D6, Developmental Studies Hybridoma Bank), rat anti-SIX1 [12] (dilution, 1:2000), guinea pig anti-SOX10 [11] (dilution, 1:20000), and mouse anti-TUBB3 (tubulin, beta 3 class III, clone TuJ1, dilution, 1:3000, Covance, Berkeley, CA) antibodies. The secondary antibodies were fluorophore (Alexa Fluor 405, 488, 546, 633 and Cy5)-labeled species-specific antibodies (dilution, 1:1000 or 1:2000) (Molecular Probes/Invitrogen and Amersham Biosciences). DAPI (4,6-diamidino-2-phenylindole, Sigma, St. Louis, MO) was used for nuclear staining. The immunofluorescence images were acquired with FV1000 (Olympus) laser confocal microscope.

Experiments were performed on at least three different embryos and the results were similar. Representative results are shown in the figures.

## Results

### Sequence characteristics of Six1-8, a major sensory neuron enhancer

In the mouse *Six1* locus, Six1-8 enhancer is located at about 9-kb upstream of the major transcription start site [17]. Homologous sequences were found not only in Tetrapoda (human, chick and *Xenopus*), but also in coelacanth and Actinopterygii (medaka and spotted gar), indicating that Six1-8 was acquired/evolved before the divergence of Sarcopterygii and Actinopterygii (Fig 1A). Similar sequences were not found in currently available genomes of elephant shark, lampreys, ascidians or amphioxus, despite the presence of exons of *Six1/2* subclass genes. The alignment of Six1-8 sequences from four tetrapod species emphasized the core conserved regions shown in Fig 1B. In the core regions, we were able to identify the binding consensus sequences for the following transcription factors: TCF/LEF (WNT/ß-catenin signaling effector), SMAD (TGF-ß/BMP signaling effector), BRN2 (POU-homeodomain protein, POU3F2), YY1 (zinc-finger protein), E-box-binding basic-helix-loop-helix (bHLH) proteins and nuclear hormone receptors (NRs). The most distinct feature was the presence of three conserved binding consensus sequences for NRs (NR1-NR3) and an additional sequence similar to NR (NR4) (Fig 1B). All the NRs were direct repeat motifs composed of two tandem AGGTCA half-sites separated by a single nucleotide (DR1: AGGTCA n AGGTCA) [57, 58]. As to the BRN2-binding consensus sequences, the identified sequences were of the GCATNNNTAAT-type, and we noticed that other POU-homeodomain proteins [BRN3A (POU4F1), BRN3B (POU4F2) and BRN3C (POU4F3)] could also bind [59–62].

### Identification of important cis-elements required for Six1-8 enhancer activity

**Fig 2:**
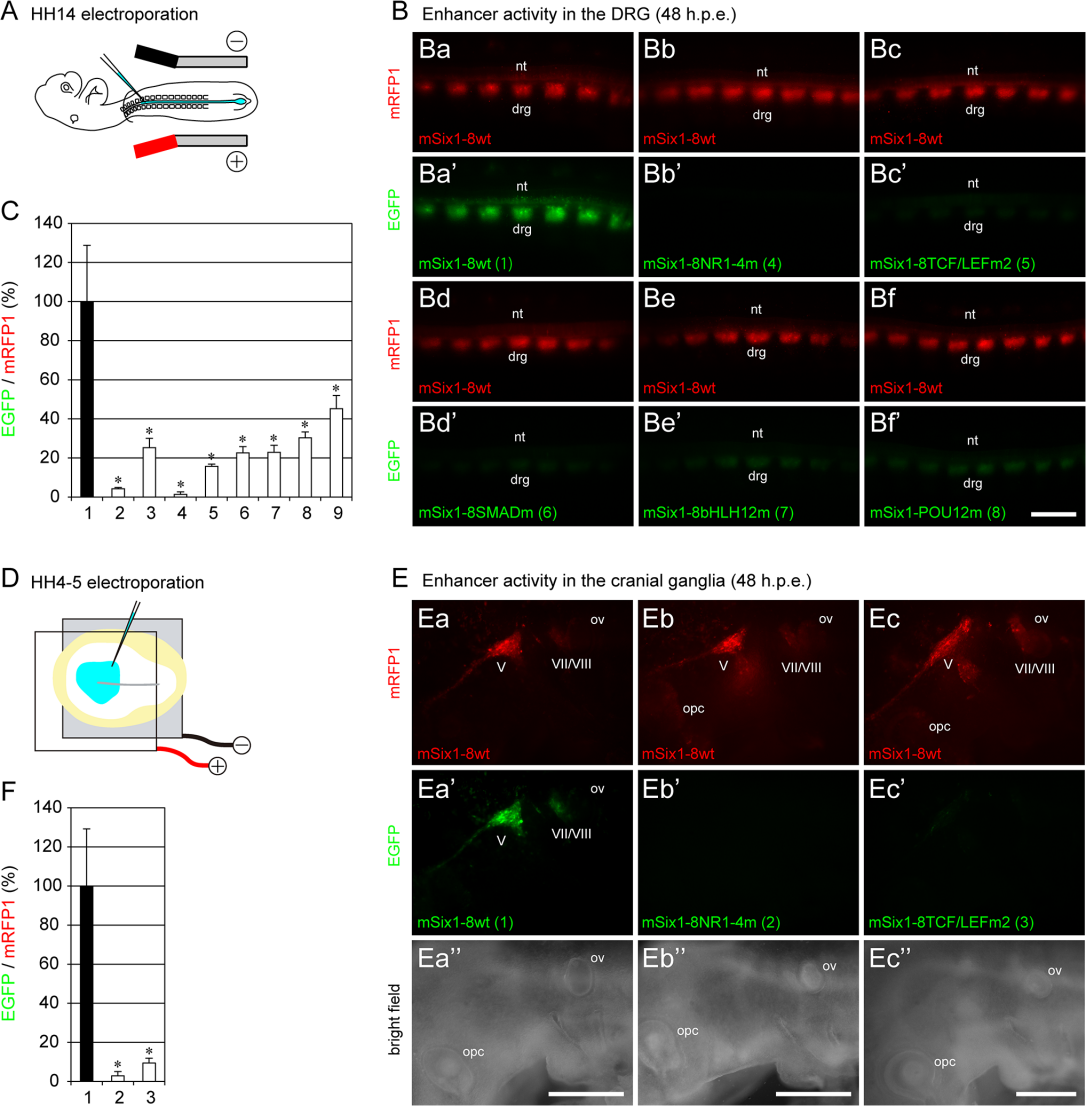
Functional analysis of mSix1-8 in chick. (A) Schematic diagram of mutation analysis of mSix1-8 enhancer in the DRG. Various mSix1-8-EGFP reporters were co-electroporated into the left side of the neural tube with the control wild-type reporter (mSix1-8wt-mRFP1) at HH14, and fluorescence intensities of EGFP and mRFP1 in the DRG were examined at 48 h.p.e. (B) Results of mutation analysis of mSix1-8 in the DRG. mSix1-8wt-mRFP1 (red) was co-electroporated with various EGFP constructs (green): mSix1-8wt (Ba, Ba'), mSix1-8NR1-4m (Bb, Bb'), mSix1-8TCF/LEFm2 (Bc, Bc'), mSix1-8SMADm (Bd, Bd'), mSix1-8bHLH12m (Be, Be') and mSix1-8POU12m (Bf, Bf'). Numbers in brackets correspond to the bar numbers (1–9) in C. The wild-type reporter (Ba-Bf, Ba') marked the DRG (drg) and weakly the neural tube (nt), while all mutations reduced EGFP levels (Bb'-Bf'). mSix1-8NR1-4m almost completely abolished mSix1-8 enhancer activity (Bb, Bb'). The image is a dorsal view of the trunk region, and anterior is to the left. (C) Quantification of the effect of various mutations on Six1-8 enhancer activity in the DRG. The relative EGFP/mRFP1 levels were calculated for each embryo by measuring five DRG, and are shown relative to the value obtained from the wild-type reporter (100%). Data are mean±SD. The relative EGFP level detected in the DRG that received reporters with various mutations was significantly lower (*p<0.001) than that of embryos received wild-type reporter. 1: wild-type (n = 7), 2: NR1m (n = 5), 3: NR2m (n = 6), 4: NR1-4m (n = 8), 5: TCF/LEFm2 (n = 6), 6: SMADm (n = 8), 7: bHLH12m (n = 6), 8: POU12m (n = 6), 9: YYm (n = 7). (D) Schematic diagram of mutation analysis of mSix1-8 enhancer in cranial ganglia. Three mSix1-8-EGFP reporters were co-electroporated into the entire epiblast with the control wild-type reporter (mSix1-8wt-mRFP1) at HH4-5, and the fluorescence intensities of EGFP and mRFP1 in the head region were examined at 48 h.p.e. (E) Mutation analysis of mSix1-8 in cranial ganglia. mSix1-8wt-mRFP1 (red) was co-electroporated with various EGFP constructs (green): mSix1-8wt (Ea, Ea', Ea''), mSix1-8NR1-4m (Eb, Eb', Eb'') and mSix1-8TCF/LEFm2 (Ec, Ec', Ec''). Numbers in brackets correspond to the bar numbers in F. The wild-type reporter (Ea-Ec, Ea') marked the trigeminal ganglion (V), geniculate (VII)/vestibuloacoustic (VIII) ganglion complex (VII/VIII) and weakly the otic vesicle (ov), while the two mutations reduced EGFP levels (Eb'-Ec'). mSix1-8NR1-4m almost completely abolished mSix1-8 enhancer activity (Eb, Eb'). mSix1-8wt shows weak enhancer activity in the posterior optic cup (opc, Eb and Ec). Each image is a lateral view of the left side of the head/neck. Anterior is to the left and dorsal is to the top. (F) Quantification of the effect of the two mutations on mSix1-8 enhancer activity in the trigeminal ganglion. The relative EGFP/mRFP1 levels were calculated for each embryo by measuring trigeminal ganglia of both sides, and are shown relative to the value obtained from the wild-type reporter (100%). Data are mean±SD. The relative EGFP level detected in trigeminal ganglia that received reporters with the two mutations was significantly lower (*p<0.001) than that of embryos received wild-type reporter. 1: wild-type (n = 5), 2: NR1-4m (n = 8), 3: TCF/LEFm2 (n = 7). drg: dorsal root ganglia, nt: neural tube, opc: optic cup, ov: otic vesicle, V: trigeminal ganglion, VII/VIII: VII/VIII ganglion complex. Scale bars: 0.5 mm.

To address the role of conserved binding consensus sequences, we introduced wild-type (ptkmRFP1-mSix1-8wt) and mutated (various ptkEGFP-mSix1-8 constructs) reporters into the neural tube of HH14 chick embryos in ovo (Fig 2A) and quantitated the effect on the expression of the reporter gene in the DRG at 48 h.p.e. (Fig 2B and 2C). The wild-type reporter activated reporter gene expression in the DRG (Fig 2Ba, 2Ba', 2Bb, 2Bc, 2Bd, 2Be and 2Bf) and weakly in the neural tube. Mutation of the putative NR-binding consensus sequences produced the most dramatic reduction in mSix1-8 enhancer activity (EGFP expression levels) in the DRG. Mutation of NR1 alone (NR1m) was associated with more than 20-fold reduction in the enhancer activity (bar 2 in Fig 2C), and mutations of all four NR sites (NR1-4m) were associated with almost no activity (Fig 2Bb’, bar 4 in C). Mutations of the binding consensus sequences for TCF/LEF (TCF/LEFm2, Fig 2Bc’), SMAD (SMADm, Fig 2Bd’), bHLH proteins (bHLH12m Fig 2Be’) and Brn2/3 (POU12m, Fig 2Bf’) were also associated with negative effects (bars 5–8 in Fig 2C). The effect of mutation of the YY1-binding consensus sequence (YYm) was associated with the mildest effects among the mutations examined, yet YYm still reduced the enhancer activity by almost 50% (bar 9 in Fig 2C).

To examine the roles of these conserved binding consensus sequences in the activation of transcription in the cranial region, including the trigeminal ganglia, by mSix1-8 enhancer, we introduced three representative reporters (wild-type, NR1-4m and TCF/LEFm2) into the epiblast of the HH4-5 chick embryos (Fig 2D), cultured *in vitro*, and then assessed the activity at 48 h.p.e. (Fig 2E and 2F). The wild-type reporter activated reporter gene expression in the trigeminal ganglion, VII/VIII ganglion complex and in the otic vesicle (Fig 2Ea, 2Ea', 2Ea'', 2Eb, 2Eb'', 2Ec and 2Ec''). NR1-4m abolished mSix1-8 enhancer activity (Fig 2Eb’, bar 2 in F) and TCF/LEFm2 also markedly reduced the enhancer activity (Fig 2Ec’, bar 3 in F). These results indicate that NR1-4 and TCF/LEFm2 sites are critical for enhancer activity both in the cranial ganglia and DRG.

**Fig 3:**
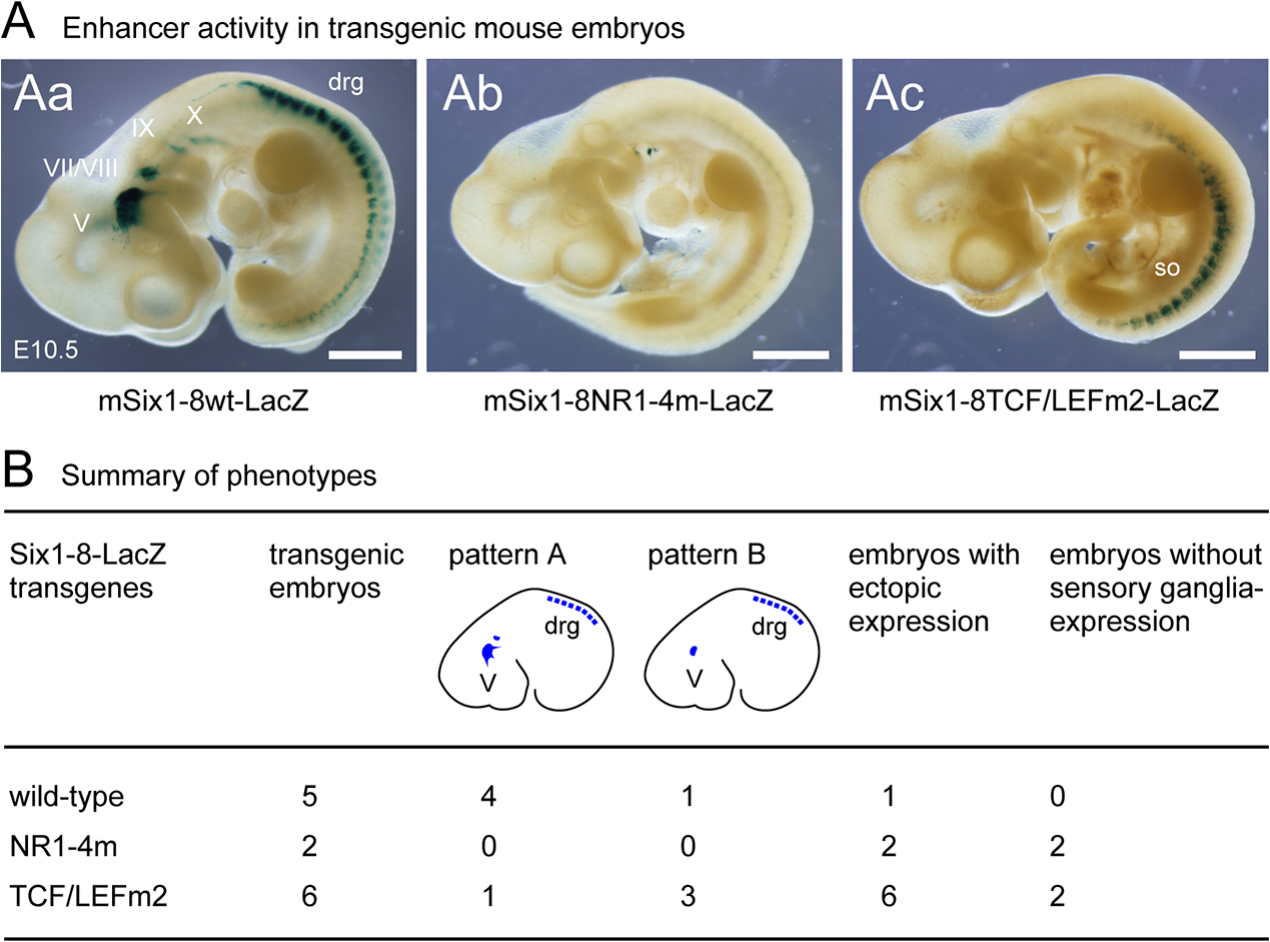
Functional analysis of mSix1-8 in mouse. (A) Mutation analysis of mSix1-8 in mouse. Wild-type (mSix1-8wt-LacZ, Aa) and mutated [(mSix1-8NR1-4m-LacZ, Ab) and (mSix1-8TCF/LEFm2-LacZ, Ac)] transgenes were used for transgenesis and ß-Gal localization was examined at E10.5. Embryos injected with the wild-type transgene showed ß-Gal activity specifically in the trigeminal ganglion, the VII/VIII ganglion complex and epibranchial placode/ganglia (Aa)[17]. However, ß-Gal activity was almost completely lost in an embryo carrying NR1-4m mutation, with the exception of a small number of ectopic ß-Gal-positive cells in the cervical area (Ab). In an embryo carrying TCF/LEFm2 mutation, ß-Gal activity was lost in the sensory organs and ganglia, with the exception of ectopic activity in the somites (Ac). drg: dorsal root ganglia, so: somites, V: trigeminal ganglion, VII/VIII: VII/VIII ganglion complex, IX: petrosal ganglion, X: nodose ganglion. Scale bars: 1 mm. (B) Summary of the phenotypes of transgenic mouse embryos. transgenic embryos: the total number of transgenic embryos obtained using each transgene, pattern A: number of embryos with ß-Gal staining pattern in which the signal in the trigeminal ganglion stands out as the major ß-Gal-positive domain (Fig 3Aa represents a typical pattern A staining), pattern B: number of embryos with ß-Gal staining in which signals in the cranial ganglia was reduced while those in the DRG were relatively unaffected, embryos with ectopic expression: number of embryos with an ectopic LacZ staining, embryos without sensory ganglia-expression: number of embryos without a LacZ staining in the sensory ganglia. Parts of the results obtained using the wild-type transgene were reported previously [17].

Finally, to confirm the importance of NR and TCF/LEF-binding consensus sequences for mSix1-8 enhancer activity in mice, we generated mouse embryos carrying wild-type and mutated mSix1-8-LacZ reporter transgenes (Fig 3). Fig 3Aa shows a ß-Gal staining pattern of the wild-type mSix1-8 transgene (ASSinsBBins-mSix1-8wt-LacZ) at E10.5 [17]. The presence of ß-Gal was highly specific to sensory ganglia (5/5 transgenic embryos, Fig 3Aa and 3B). At this stage, the signal in the trigeminal ganglion was intense and easily stood out as the major ß-Gal-positive domain in most of the embryos (4/5 transgenic embryos, pattern A, Fig 3Aa and 3B). As expected, NR1-4m markedly reduced ß-Gal activity both in the head and trunk (2/2 transgenic embryos, Fig 3Ab and 3B). In contrast, TCF/LEFm2 resulted in complete loss of ß-Gal activity in sensory ganglia (2/6 transgenic embryos, Fig 3Ac and 3B), disruption of the aforementioned wild-type pattern (3/6 transgenic embryos, pattern B, Fig 3B) and ectopic activation of the transgene (6/6 transgenic embryos, Fig 3B). The result that both NR and TCF/LEF-binding consensus sequences were required for normal mSix1-8 activity was consistent with the data obtained in chick. Thus, while it is difficult to control the number/site of transgenes integrated into the individual embryos by pronuclear injection and to directly compare ß-Gal expression levels among different embryos/transgenes, the results clearly emphasize the importance of both sites in the regulation of mSix1-8.

### Establishment of mSix1-8-NLSCre mouse line

**Fig 4:**
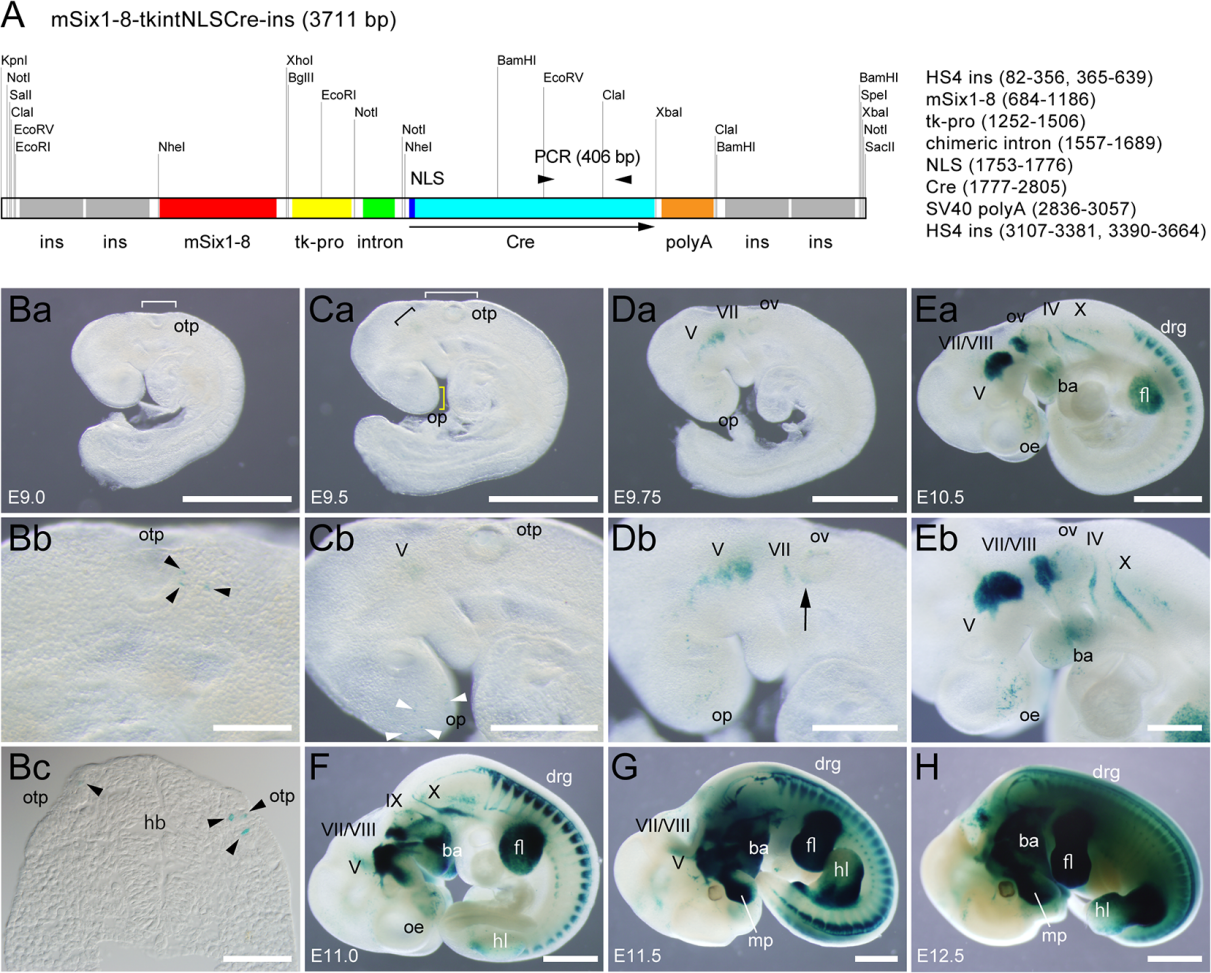
Pattern and specificity of mSix1-8-NLSCre-mediated recombination in embryos. (A) The structure of a transgene used to generate the mSix1-8-NLSCre transgenic mouse line. mSix1-8 (538 bp) is placed upstream of the tkintron (the HSV thymidine kinase gene promoter and chimeric intron) to drive the expression of NLSCre. The polyA signal is from SV40. The entire expression unit is flanked by two tandem copies of the core region of the HS4 insulator (ins). The positions of the genotyping PCR primers (arrowheads) and the size of the PCR product (406 bp) are shown. Selected restriction sites are also indicated. (B-H) Localization of ß-Gal-positive cells in mSix1-8-NLSCre/R26R-LacZ double transgenic embryos. At E9.0 (Ba), the earliest sign of the appearance of ß-Gal-positive cells was detected in the otic pit region (white square bracket). A close-up view (Bb) shows signals in three scattered cells (black arrowheads) in the otic pit. A section through the posterior part of the otic pit confirmed the presence of ß-Gal-positive cells in the pits of both sides (Bc). At E9.5 (Ca), ß-Gal-positive cells were noted in the developing trigeminal ganglion (black square bracket) and olfactory placode (yellow square bracket) in addition to the otic pit (white square bracket). A close-up view (Cb) shows signals in scattered cells in and around the olfactory placode (white arrowheads). At E9.75 (Da), signals were detected in the developing geniculate ganglion. In a close-up view (Db) showed signals in the ventral portion of the otic vesicle (black arrow). At E10.5 (Ea), clear signals were detected in all the cranial sensory ganglia (V, VII, VIII, IX and X), cells in and around the olfactory epithelium (Eb, a close-up view) and in the DRG. At E11.0 (F), the intensity of the signals in the sensory organs became stronger. Signals were also found in the mesenchyme of forelimb bud (Ea, F-H), hindlimb bud (F-H), branchial arches (Ea, Eb, F-H), and the maxillary process (F-H). In all panels of whole mount embryos, anterior is to the left, dorsal is to the top, and all panels are lateral views. In the transverse section shown in Bc, dorsal is to the top. ba: branchial arches, drg: dorsal root ganglia, fb: forebrain, fl: forelimb bud, hb: hindbrain, hl: hindlimb bud, mp: maxillary process, oe: olfactory epithelium, op: olfactory placode, otp: otic pit, ov: otic vesicle, V: trigeminal ganglia, VII: geniculate ganglia, VII/VIII: VII/VIII ganglion complex, IX: petrosal ganglion, X: nodose ganglion. Scale bars: 2 mm (H), 1 mm (Ba, Ca, Da, Ea, F, G), 0.2 mm (Bb), 0.5 mm (Cb, Db, Eb), 0.1 mm (Bc).

The sequence and functional characteristics of mSix1-8 described above prompted us to use the enhancer as a tool to drive the expression of transgenes in a sensory organ-specific manner. For this purpose, we first generated a new transgenic mouse line (mSix1-8-NLSCre) that carries a transgene (Fig 4A) for the expression of NLSCre under the control of the mouse Six1-8 enhancer. To examine the pattern and efficacy of Cre-mediated recombination, the mSix1-8-NLSCre line was crossed with the R26R-LacZ reporter line [53] and mSix1-8-NLSCre/R26R-LacZ double transgenic embryos were stained for ß-Gal activity (Fig 4B–4H). Positive ß-Gal staining appeared at E9.0 in a few scattered cells in and around the otic pit (Fig 4Ba and 4Bb). The exact position of the early ß-Gal-positive cells was different among embryos and was not bilaterally symmetrical, but usually associated with the pit (Fig 4Bc). At E9.5, ß-Gal-positive cells were also detected in the developing trigeminal ganglion (V) and scattered cells were found in the olfactory placode (OP) and in the surrounding ectoderm in addition to the aforementioned otic area (Fig 4Ca and 4Cb). The ß-Gal signals gradually intensified, and at E9.75, positive cells were detected in the geniculate ganglion (VII) (Fig 4Da and 4Db). ß-Gal-positive cells were also evident in the ventral portion of the otic vesicle (Fig 4Db, black arrow), which most likely represent sensory neurons that form the vestibuloacoustic ganglion. At E10.5, clear ß-Gal signals were detected in the developing vestibuloacoustic (VIII) ganglion that formed the VII/VIII ganglion complex, rest of the epibranchial sensory ganglia [petrosal (IX) and nodose (X) ganglia], scattered cells in and around the olfactory epithelium (OE) and the DRG in the trunk (Fig 4Ea and 4Eb). Thus, the use of mSix1-8-NLSCre transgenic mouse allowed us to induce recombination in all the cranial sensory ganglia and DRG at E10.5. Such ß-Gal signals in the sensory organs became more intense at E11.0 (Fig 4F). Additional ß-Gal signals were also detected in the mesenchyme of the fore- and hindlimb buds, branchial arches and the maxillary process (Fig 4Ea, 4Eb and 4F–4H).

**Fig 5:**
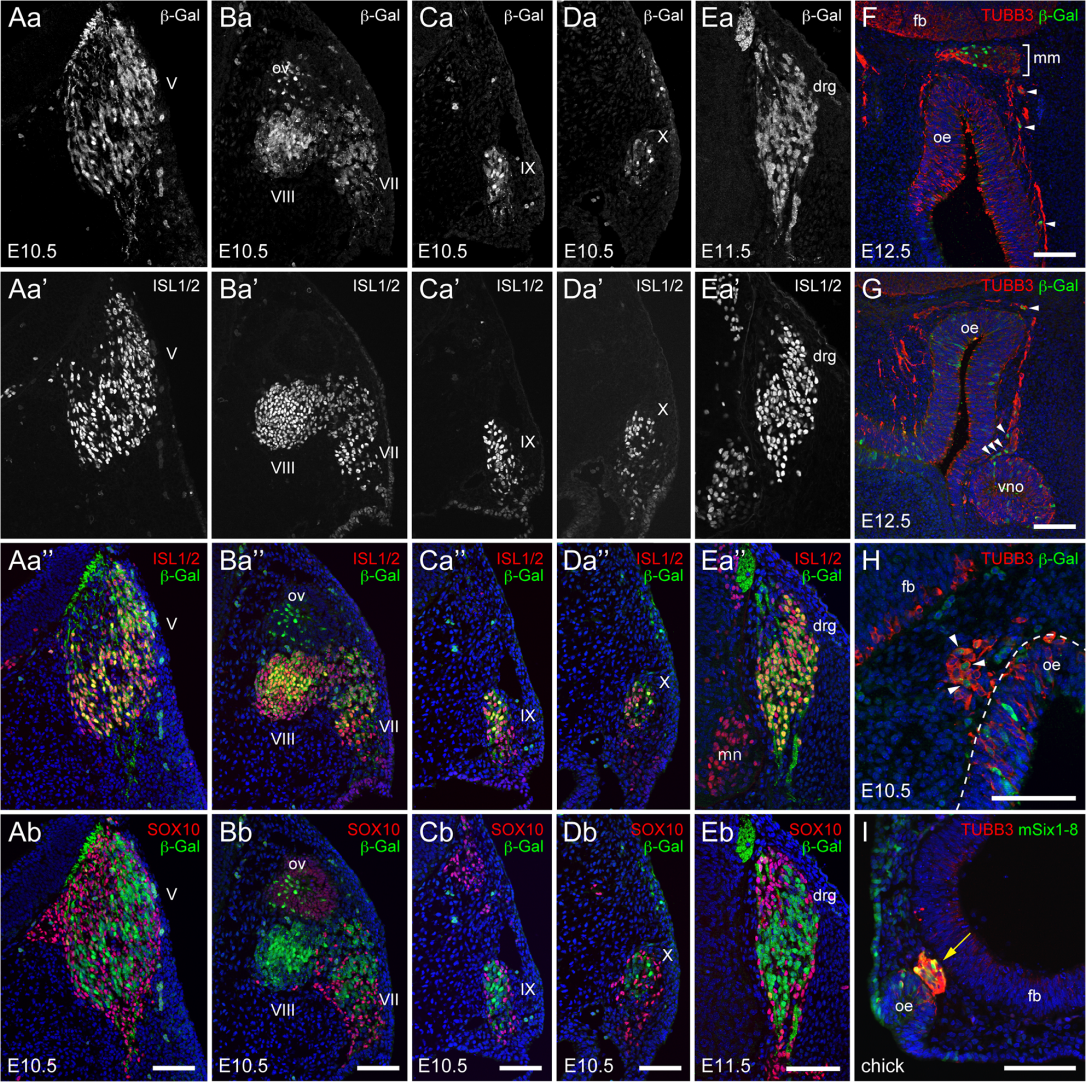
mSix1-8-NLSCre induces sensory neuron-specific recombination in mouse embryos. (A-I) Immunofluorescence analysis of Cre-mediated recombination in mSix1-8-NLSCre/R26R-LacZ double transgenic embryos. Distribution of ß-Gal was examined at E10.5 (A-D, H), E11.5 (E) and E12.5 (F, G). At E10.5, ß-Gal (Aa) co-localized with a neuron marker ISL1/2 (Aa') in the trigeminal ganglion (yellow signals in Aa''), geniculate-vestibuloacoustic ganglion complex (Ba, Ba', Ba''), petrosal ganglion (Ca, Ca', Ca'') and nodose ganglion (Da, Da', Da'') in transverse sections through the cervical area. In contrast, the same sections stained for a glial marker SOX10 showed no overlap of ß-Gal (green)-positive cells with SOX10 (red)-positive cells in all cranial sensory ganglia (Ab, Bb, Cb, Db). At E11.5, ß-Gal (Ea) also co-localized with ISL1/2 (Ea') in the DRG (yellow signals in Ea'') in a transverse section through the trunk region but not with SOX10 on the same section (Eb). In the frontal sections through the OE at E12.5 (F, G), ß-Gal (green)-positive cells were detected not only in the OE (F, G) but also in the vomeronasal organ (G) and in the surrounding mesenchyme along the TUBB3 (red)-positive axons of the olfactory and vomeronasal sensory neurons (white arrowheads). Many ß-Gal-positive cells were also found in the "migratory mass (white square bracket)" comprising placode-derived migratory cells and axons of olfactory sensory neurons located ventral to the forebrain (F). (H) At E10.5, ß-Gal (green)-positive cells were found in the OE (demarcated by white dotted line) and in an aggregate of TUBB3-positive cells (white arrowheads) located next to the OE. (I) Immunofluorescence analysis of Six1-8 enhancer activity in chick. Frontal section through the olfactory pit of a representative chick embryo at 48 h.p.e. EGFP (green) derived from pT2A-BB-mSix1-8-EGFP was detected in the OE and in an aggregate of TUBB3 (red)-positive cells (yellow arrow) located subjacent to the OE, a likely avian homolog of the rodent migratory mass. DAPI was used for nuclear staining (blue, Aa'', Ab, Ba'', Bb, Ca'', Cb, Da'', Db, Ea'', Eb, F-I). In all panels, dorsal is to the top and midline is to the left (A-E, H) or right (F, G, P). drg: dorsal root ganglia, fb: forebrain, mm: migratory mass, oe: olfactory epithelium, ov: otic vesicle, vno: vomeronasal organ, V: trigeminal ganglia, VII: geniculate ganglia, VIII: vestibuloacoustic ganglion, IX: petrosal ganglion, X: nodose ganglion. Scale bars: 0.1 mm (A-I).

Next, immunofluorescence analysis was performed to assess the cell type specificity of Cre-mediated recombination. As shown in Fig 5A–5H, ß-Gal protein was detected in most of the cells positive for sensory neuron marker ISL1/2 in the trigeminal ganglion (Fig 5Aa–5Aa''), VII/VIII-ganglion complex (Fig 5Ba–5Ba''), petrosal ganglion (Fig 5Ca–5Ca''), nodose ganglion (Fig 5Da–5Da'') and DRG (Fig 5Ea–5Ea''). In contrast, ß-Gal-positive cells were distinct from cells positive for the neural crest/glial marker SOX10 (Fig 5Ab, 5Bb, 5Cb, 5Db and 5Eb), indicating specific ß-Gal production in sensory neurons. Localization of neuron-specific ß-Gal was consistent with the expression pattern of endogenous SIX1 reported previously [11, 12]

### mSix1-8-NLSCre line can direct DNA recombination in olfactory tissues

As described above, ß-Gal activity was detected in scattered cells in the OP as early as E9.5 (Fig 4Ca and 4Cb). The ß-Gal-positive cells increased in number but remained confined to a subset of cells (Fig 4Da and 4Db, S1 Fig) and they never covered the entire placode/ectoderm marked by SIX1 during the following stages (Fig 4E–4H). In the E12.5 olfactory tissue, cells positive for ß-Gal were detected not only in the OE but also in the vomeronasal organ and in the surrounding mesenchyme along the TUBB3-positive axons from the olfactory and vomeronasal sensory neurons (Fig 5F and 5G). Interestingly, many ß-Gal-positive cells were found in the so-called "migratory mass [6, 63, 64]", a cellular aggregate comprising OP/OE-derived migratory cells and axons of olfactory sensory neurons within the mesenchyme ventral to the forebrain (Fig 5F). This suggested Cre-mediated recombination in the olfactory pioneer neurons at earlier developmental stages. As expected, ß-Gal was detected in TUBB3-positive neurons located in the OE and in the cellular aggregate adjacent to the OE at E10.5 (Fig 5H). The presence of ß-Gal-positive cells in the olfactory tissues was unexpected because there was no reproducible ß-Gal staining in the olfactory or the surrounding tissues of E10.5 transgenic mouse embryos carrying mSix1-8wt-LacZ (Fig 3Aa). Accordingly, we next investigated whether Six1-8 has inherent and conserved ability to activate transcription in the OP/OE. For clear detection of olfactory enhancer activity in the chick embryo, a reporter transgene (pT2A-BB-mSix1-8-EGFP) was introduced into the HH4-5 epiblast using the Tol2 transposon-mediated integration system [49, 50]. As shown in Fig 5I, EGFP expression was detected at 48 h.p.e. in the OE and in an aggregate of TUBB3-positive cells, a likely avian homolog of the rodent migratory mass mentioned above, located subjacent to the OE. These results suggest that Six1-8 is a conserved enhancer for olfactory tissues, particularly neurons that emigrate during early stages from the OP/OE and later localize along the axons of olfactory sensory/vomeronasal neurons.

## Discussion

In this study, we analyzed the sequence and functional characteristics of Six1-8, a sensory ganglia-specific *Six1* enhancer. We identified multiple transcription factor binding consensus sequences critical for tissue-specific Six1-8 enhancer activity by sequence comparison and *in vivo* enhancer analyses. In addition, by using Six1-8 enhancer as a tool, we have established a new transgenic mouse line that exhibits Cre recombinase activity specifically in the sensory neurons of all cranial sensory ganglia and DRG and in a subset of neurons derived from the OP/OE.

### Regulation of Six1-8 enhancer activity by upstream factors and signals

The loss of *Six1* damages sensory development from the OP and otic vesicle and affects neurogenesis in the distal part of the epibranchial ganglia [5–10]. Mice deficient in both *Six1* and *Six4* show a more severe phenotype characterized by lack of OP [9], presence of a small otic vesicle (data not shown), and abnormal development of the trigeminal ganglion and DRG [8, 9, 11, 12], reflecting the functions of both genes during early development. The present study demonstrated that mutations of binding consensus sequences for several transcription factors have clear negative effects on Six1-8 (Figs 1–3).

Among the potential binding sequences, the most important were conserved NR-binding sites. All the identified Six1-8 NR-sites were of DR1-type, suggesting that RXR homodimers or heterodimers among RXR, RAR, PPAR and COUP-TFI (NR2F1)/COUP-TFII (NR2F2) are the candidate binding factors [57, 65, 66]. Retinoic acid (RA) signaling activity was detected in the OE [67], all cranial sensory ganglia and DRG [68]. Indeed, the involvement of RA signaling has been reported for the otic vesicle induction [69], otic neurogenesis [70, 71] and olfactory neurogenesis [72]. NR2F1/NR2F2 are known to repress RA-mediated transcription activation and can antagonize RA-induced neuronal differentiation [73, 74].

There are transcription factors other than NRs that can bind important cis-elements identified in Six1-8 and have known roles during early stages of sensory neurogenesis. Canonical Wnt/ß-catenin signaling that acts through nuclear effector TCF/LEFs is an established key player in the sensory development from the neural crest [75, 76]. Wnt signaling activity were also detected in the trigeminal ganglion [77], and blocking of the canonical Wnt pathway leads to the failure of the head ectoderm to adopt or maintain ophthalmic trigeminal placode fate [78]. A group of bHLH proteins control the initial phase of sensory neurogenesis in the cranial and trunk regions [79, 80]. After the neurogenic phase, sensory neurons co-express the pan-sensory homeodomain protein ISL1 and POU-homeodomain factor BRN3A [28, 81]. *Six1* expression driven by Six1-8 (Figs 4 and 5) is therefore similar in specificity and timing to ISL1 and BRN3A. BRN3A as well as BRN3B are expressed in the trigeminal ganglion and DRG [59–62]. The lack of BRN3A affects the differentiation and survival of trigeminal neurons, while the lack of BRN3A and/or BRN3B affects the specification and projection of DRG neurons [82, 83]. The above described factors and signals are potentially relevant to *Six1* regulation through Six1-8. However, it should be borne in mind that the more direct methods are needed to prove Six1-8 is regulated by any of these factors in vivo. The present study provided the basis for the further study that aims to directly identify binding factors and elucidate a complete picture of *Six1* regulation in sensory neurons.

### Conservation and diversity of *Six1* expression in sensory neurons

SIX1 is expressed specifically in sensory neurons in mouse embryos [8, 11] and the present study demonstrated that the expression is primarily controlled by Six1-8. As shown in Fig 1, Six1-8 is conserved not only in tetrapods but also in Sarcopterygii and Actinopterygii. Interestingly, zebrafish retains a sequence similar (e.g. >50% identity over 100 bp) to Six1-8 [17] but the sequence lacks binding consensus sequences for key transcription factors (data not shown), and *six1a* and *six1b*, two paralogous zebrafish *Six1* genes, are not expressed in the trigeminal ganglion or in the DRG [18, 84, 85]. Since Six1-8 is found in other teleosts, such as medaka and stickleback (data not shown), it would be interesting to examine the phylogenetic changes in the roles of *Six1* and Six1-8 in sensory development (including developmental transition from RB to DRG neurons) in teleosts. Another issue of interest is how deep the connection between sensory neurogenesis and *Six1* and/or Six1-8 is conserved during evolution. We suggest that the connection was established before the divergence of Sarcopterygii and Actinopterygii. Although sequences similar to Six1-8 were not detected in lampreys, amphioxus or ascidians (data not shown), further studies are needed to investigate the regulation of epidermal sensory neurons that express *AmphiSix1/2*, the sole amphioxus *Six* gene of *Six1/2* subclass [86].

### mSix1-8-NLSCre should be useful for study of sensory development

In the mSix1-8-NLSCre line, Cre recombinase is expressed at very early stages in the cranial sensory ganglia and DRG. The specificity of Cre-mediated recombination in the sensory neurons was confirmed in the trigeminal, vestibuloacoustic-geniculate, petrosal and nodose ganglia and DRG (Figs 4 and 5). On the other hand, Cre expressing lines driven by regulatory elements of *Brn3a* [26, 87], *Neurog1* [88], *Neurog2* [89], *Isl1* [90] and *Phox2b* [91] loci all induce recombination in the central nervous system as well as in the peripheral sensory neurons. Previous studies showed that the isolated CREST3 element of *Isl1* exhibits sensory neuron-specific enhancer activity similar to Six1-8 [27]. Other studies identified several other DNA elements with similar property at E11.5 (e.g. http://enhancer.lbl.gov/ [92]). However, with the exception of CreN752 line that expresses the N-terminal half of Cre fused to Intein under the control of hs752 element [93], Cre expressing lines utilizing those enhancers have not been described or to our knowledge are not yet available. Thus, the mSix1-8-NLSCre line that does not induce recombination in the central nervous system should be valuable for tracing the central projection of sensory neurons and to examine the effect of gene knockout on central targets of sensory neurons.

Another feature of the mSix1-8-NLSCre line was the occurrence of Cre-mediated recombination in the OP as early as E9.5 (Figs 4 and 5). The result appears to contradict the finding that ß-Gal activity driven directly by Six1-8 was barely detectable in the OE or in the adjacent mesenchyme at E10.5 (Fig 3). There are two possible explanations for this finding: i) the expression of Cre in the OP/OE merely reflects the influence of a transgene integration site, ii) Six1-8 is a transient olfactory enhancer with weak activity and/or cell-type specific activity and ß-Gal-positive cells observed in the following developmental stages are descendants of these cells. The fact that Six1-8 enhancer is active in the OE and neurons in the "migratory mass" in stably electroporated chick embryos (Fig 5) support the latter view. We speculate that the time window where Six1-8 acts as an olfactory enhancer is short and limited to the E9.5–10.5 period in mice. Then, in which type of cells Six1-8 is activated? The preferential localization of ß-Gal-positive neurons in the "migratory mass" at E10.5 and E12.5 (Fig 5) suggests that Cre is transiently turned on in the precursors of migratory neurons. In the mouse OP lineage, *Six1* is expressed in the entire placode (from E9.0, S1 Fig), OE and olfactory/vomeronasal sensory neurons [6, 9, 10], and we have previously reported that Six1-21 is a major olfactory enhancer involved in such expression. As such, early onset of SIX1 expression or additional dose of SIX1 dictated by Six1-8 enhancer in a subset of OP cells may result in qualitative difference among OP lineage cells and which might be relevant to the specification or generation of migratory neurons. Finally, since mSix1-8-NLSCre induces recombination only in a subset of OP cells, the use of this line should allow analysis of migration pattern, morphological changes [94, 95] and the effect of cell ablation or over-expression at the level of individual OP/OE cells.

## Supporting Information

S1 Fig ß-Gal is detected in a subset of cells in the olfactory placode. (A-D) Histochemical analysis of Cre-mediated recombination in the E9.75 mSix1-8-NLSCre/ R26R-LacZ double transgenic embryo shown in Fig 4Da. Distribution of ß-Gal was examined in the frontal sections of the head. ß-Gal activity (A) and protein (C) are detected in a subset of cells in the thickened olfactory placode (demarcated by white dotted line) marked by a high level of SIX1 (C). ß-Gal protein is co-localized with SIX1 in the OP (D). Cells positeve for ß-Gal protein or activity are highlighted by white arrowheads (A-D). A low level of SIX1 is also detected in the mesenchyme between the OP and forebrain (C). The X-Gal stained embryo was cut into 14-μm thick sections. The primary antibodies: rabbit anti-ß-Gal (dilution, 1:5000, Covance), genea pig anti-SIX1 (dilution, 1:5000, [11]). The secondary antibodies: fluorophore (Alexa Fluor 488 and 546)-labeled species-specific antibodies (dilution, 1:1000) (Molecular Probes/Invitrogen and Amersham Biosciences). DAPI was used for nuclear staining (D). The image of ß-Gal staining (A) was obtained with a standard microscope (BX51, Olympus) and the immunofluorescence images (B-D) were acquired with a laser confocal microscope (FV1000, Olympus). In all panels, dorsal is to the top. fb: forebrain, me: mesenchyme, op: olfactory placode. Scale bar: 0.2 mm.
(PDF)
