# Supplementary material for: Activation of Six1 Expression in Vertebrate Sensory Neurons
Source: PLoS One. 2015 Aug 27;10(8):e0136666. doi: 10.1371/journal.pone.0136666 (PMC4551851; doi:10.1371/journal.pone.0136666)
Supplement: S1 Fig — (A-D) Histochemical analysis of Cre-mediated recombination in the E9.75 mSix1-8-NLSCre/ R26R-LacZ double transgenic embryo shown in Fig 4Da. Distribution of ß-Gal was examined in the frontal sections of the head. ß-Gal activity (A) and protein (C) are detected in a subset of cells in the thickened olfactory placode (demarcated by white dotted line) marked by a high level of SIX1 (C). ß-Gal protein is co-localized with SIX1 in the OP (D). Cells positeve for ß-Gal protein or activity are highlighted by white arrowheads (A-D). A low level of SIX1 is also detected in the mesenchyme between the OP and forebrain (C). The X-Gal stained embryo was cut into 14-μm thick sections. The primary antibodies: rabbit anti-ß-Gal (dilution, 1:5000, Covance), genea pig anti-SIX1 (dilution, 1:5000, [11]). The secondary antibodies: fluorophore (Alexa Fluor 488 and 546)-labeled species-specific antibodies (dilution, 1:1000) (Molecular Probes/Invitrogen and Amersham Biosciences). DAPI was used for nuclear staining (D). The image of ß-Gal staining (A) was obtained with a standard microscope (BX51, Olympus) and the immunofluorescence images (B-D) were acquired with a laser confocal microscope (FV1000, Olympus). In all panels, dorsal is to the top. fb: forebrain, me: mesenchyme, op: olfactory placode. Scale bar: 0.2 mm. (PDF) [file pone.0136666.s001.pdf]

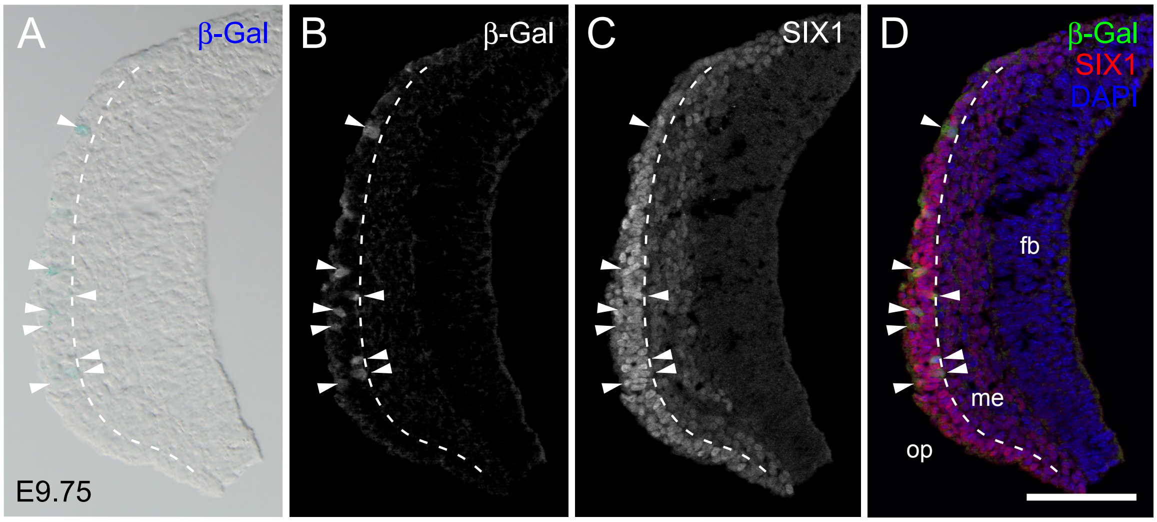

S1 Fig. β-Gal is detected in a subset of cells in the olfactory placode.
(A-D) Histochemical analysis of Cre-mediated recombination in the E9.75 mSix1-8-NLSCre/R26R-LacZ double transgenic embryo shown in Fig. 4Da. Distribution of β-Gal was examined in the frontal sections of the head. β-Gal activity (A) and protein (C) are detected in a subset of cells in the thickened olfactory placode (demarcated by white dotted line) marked by a high level of SIX1 (C). β-Gal protein is co-localized with SIX1 in the OP (D). Cells positeve for β-Gal protein or activity are highlighted by white arrowheads (A-D). A low level of SIX1 is also detected in the mesenchyme between the OP and forebrain (C). The X-Gal stained embryo was cut into 14-µm thick sections. The primary antibodies: rabbit anti-β-Gal (dilution, 1:5000, Covance), genea pig anti-SIX1 (dilution, 1:5000, [1]). The secondary antibodies: fluorophore (Alexa Fluor 488 and 546)-labeled species-specific antibodies (dilution, 1:1000) (Molecular Probes/Invitrogen and Amersham Biosciences). DAPI was used for nuclear staining (D). The image of β-Gal staining (A) was obtained with a standard microscope (BX51, Olympus) and the immunofluorescence images (B-D) were acquired with a laser confocal microscope (FV1000, Olympus). In all panels, dorsal is to the top. fb: forebrain, me: mesenchyme, op: olfactory placode. Scale bar: 0.2 mm.
